# *Aggregatibacter actinomycetemcomitans* Cytolethal Distending Toxin-Induces Cell Cycle Arrest in a Glycogen Synthase Kinase (GSK)-3-Dependent Manner in Oral Keratinocytes

**DOI:** 10.3390/ijms231911831

**Published:** 2022-10-05

**Authors:** Bruce J. Shenker, Lisa P. Walker, Ali Zekavat, Jonathon Korostoff, Kathleen Boesze-Battaglia

**Affiliations:** 1 Department of Basic and Translational Sciences, School of Dental Medicine, University of Pennsylvania, Philadelphia, PA 19104, USA; 2 Department of Periodontics, School of Dental Medicine, University of Pennsylvania, Philadelphia, PA 19104, USA

**Keywords:** cytolethal distending toxin, *Aggregatibacter actinomycetemcomitans*, cell cycle arrest, GSK3, apoptosis, DNA damage response, epithelial cells

## Abstract

Cytolethal distending toxins (Cdt) are produced by a diverse group of pathogens. One Cdt-producing organism, *Aggregatibacter actinomycetemcomitans*, plays a critical role in the pathogenesis of a unique form of periodontitis, formerly referred to as localized aggressive periodontitis. The active Cdt subunit, CdtB, is a potent phosphatidylinositol (PI) 3,4,5-triphosphate phosphatase capable of inducing PI-3-kinase signaling blockade, a requisite for Cdt-induced toxicity in lymphocytes. In this study, we extended our observations to include the oral keratinocyte response to *Aa*Cdt using cell lines and primary gingival keratinocytes. All three exhibited G2/M arrest when exposed to *Aa*Cdt toxin within 24 h. Toxin-treated cells exhibited reduced levels of pAkt and pGSK3β within 6 h. Pre-treatment with GSK3β kinase inhibitors, LY2090314, CHIR99021 and Tideglusib, abrogated Cdt-induced G2/M arrest. None of the oral epithelial cells exhibited evidence of apoptosis. Cells remained arrested in the G2/M phase for at least 72 h without evidence of DNA damage response activation (H2AX phosphorylation). Cdt-treated cells displayed increased phosphorylation of the cyclin dependent kinase 1 (CDK1); moreover, the GSK3 inhibitors blocked this increase and reduced total CDK1 levels. This study further clarifies the potential mechanism(s) contributing to Cdt toxicity and toxin-mediated pathogenesis.

## 1. Introduction

The cytolethal distending toxin (Cdt) was first described by Johnson and Lior [1,2] in culture filtrates obtained from clinical isolates of *Campylobacter* spp (including *C. jejuni, C. coli, C. fetus, C. lari*), *Escherichia coli* and *Shigella dysenteriae*. Specifically, Cdt-containing filtrates were reported to adversely affect a number of epithelial cell lines including CHO cells, Vero cells, HeLa cells and Hep-2 cells by inducing progressive distention eventually leading to loss of cell vitality. Cdts are now known to represent a conserved and highly distributed family of putative virulence factors produced by a diverse group of more than 30 γ- and ε-Proteobacteria which are responsible for chronic infections and inflammatory diseases typically affecting mucocutaneous tissue (reviewed in [3]). Human pathogens that produce Cdt include: an oral pathogen, a genital pathogen responsible for sexually transmitted chancroid, gastric pathogens and carcinogenic pathogens [4,5,6,7,8,9,10,11,12,13]. Regardless of the microbial source, all Cdts cause similar effects on proliferating cells: cell cycle arrest (most typically G2/M arrest) and eventual cell death mediated by activation of the apoptotic cascade [5,7,14,15,16,17,18,19,20,21,22]. More recent observations suggest that Cdt is capable of inducing functional alterations in the absence of apoptosis in non-proliferating cell populations [14,23,24].

The Cdts are encoded by three genes, *cdtA*, *cdtB*, and *cdtC*, arranged as an operon which encodes three polypeptides designated as CdtA, CdtB and CdtC with apparent molecular masses of 23–30, 28–32 and 19–20 kDa, respectively. The three gene products associate with one another to form a heterotrimeric holotoxin [7,14,25,26,27,28]. In contrast to all other bacteria expressing a Cdt, *Salmonella enterica* serotype Typhi (*S. Typhi*) lacks genes encoding CdtA and CdtC [9,29,30,31]. In typhoid toxin, CdtB is expressed as part of a holotoxin with a unique stoichiometry, A2B5. This toxin contains two different A subunits (CdtB and PltA) which are homologs of the A subunit of Cdt and pertussis toxin, respectively, and a pentameric B subunit (composed of 5 PltB peptides), a homolog of the B subunit of pertussis toxin.

Internalization of the active subunit, CdtB, leads to irreversible cell cycle arrest and eventual cell death via activation of the apoptotic cascade in a number of target cells, including non-oral epithelial cells. These toxic effects were originally attributed to CdtB’s ability to function as a DNase and thereby cause DNA damage which in turn was proposed to lead to G2/M arrest and death [32,33,34,35,36,37,38,39,40,41]. Over the past several years, our studies have suggested a new paradigm to account for *A. actinomycetemcomitans* Cdt-mediated (*Aa*Cdt) toxicity which is based upon a novel molecular mode of action for CdtB. In this regard, we have demonstrated that CdtB is a potent lipid phosphatase capable of converting the signaling lipid phosphatidylinositol (PI)-3,4,5-triphosphate (PIP3) to PI-3,4-diphosphate [23,42,43,44,45]. Moreover, our investigations have shown that the ability of CdtB to function as a PIP3 phosphatase enables this subunit to intoxicate cells via blockade of the PI-3K signaling pathway. Indeed, we have demonstrated that the toxic effects of Cdt on lymphocytes, macrophages and mast cells result in PI-3K signaling blockade characterized by decreases in PIP3 leading to concomitant reductions in the phosphorylation status of downstream kinase targets: Akt and glycogen synthase kinase 3β (GSK3β). Additionally, we have established that induction of G2/M arrest is dependent upon GSK3β activation while apoptosis is additionally reliant upon the cycle-dependent kinase inhibitor known as CDK-interacting protein 1 (Cip1) and wild-type p53-activated fragment 1 [(WAF1), p21^CIP1/WAF1^] [46]. These observations have been extended to Cdts produced by *Haemophilus ducreyi* (*Hd*Cdt) as well as *Campylobacter jejuni* (*Cj*Cdt) and demonstrated that CdtB contained in these toxins are also potent PIP3 phosphatases capable of inducing PI-3K blockade in lymphocytes [47].

We have now extended our studies to include assessment of *Aa*Cdt on human oral keratinocytes, both immortalized cell lines and primary cells. Oral keratinocytes share the properties of other cells with respect to being susceptible to Cdt-induced G2/M arrest. However, unlike most other cells, oral epithelial cells were not susceptible to toxin-induced apoptosis. In contrast to other epithelial cells, oral keratinocytes also did not exhibit evidence of toxin-induced DNA damage response (DDR) activation at levels of toxin exposure that resulted in cell cycle arrest. Similar to other cells we have studied, cell cycle arrest promoted by Cdt was observed to be dependent upon CdtB’s PIP3 phosphatase activity and, in particular, GSK3β activation. Moreover, we now demonstrate that cyclin-dependent kinase 1 (CDK1) phosphorylation and protein levels are regulated by GSK3β.

## 2. Results

**Figure 1:**
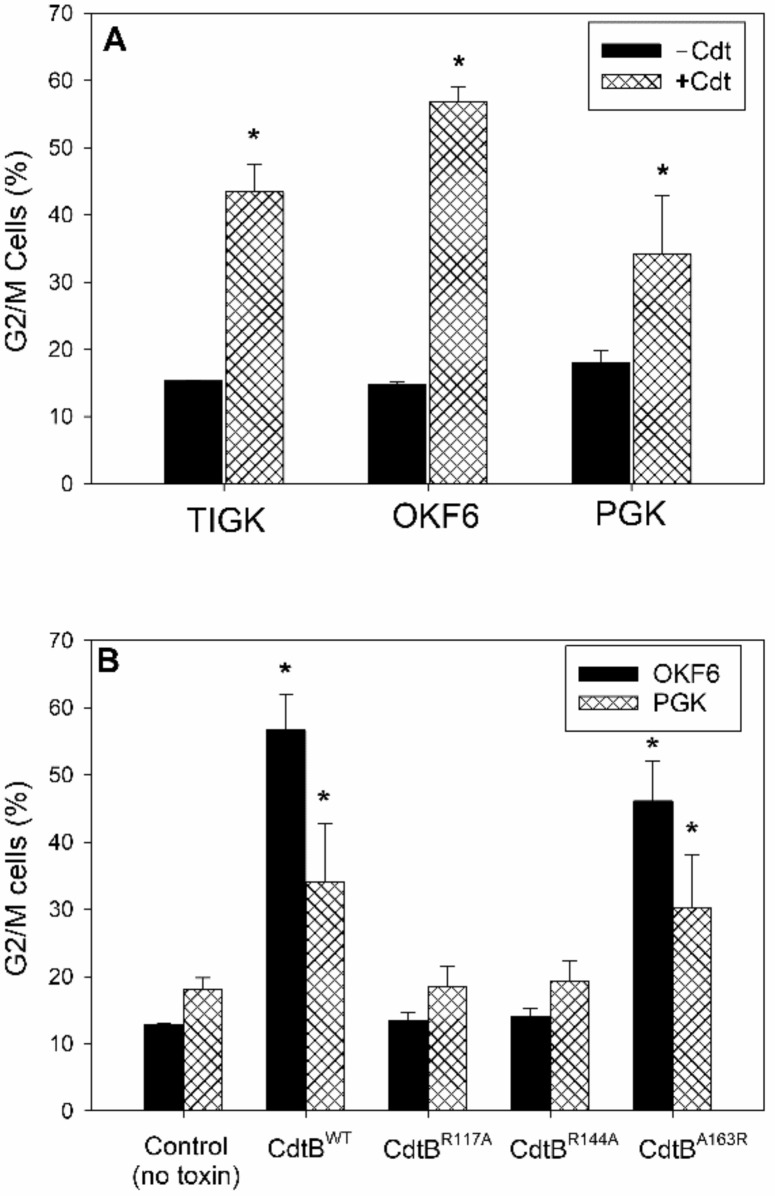
Cdt induces cell cycle arrest in human oral keratinocytes. Panel (**A**) shows the effect of Cdt on the accumulation of G2/M cells when the immortalized cell lines, TIGK and OKF6, were challenged with 2 and 10 pg/mL Cdt, respectively, for 24 h; PGK cells were challenged with 10 pg/mL Cdt for 72 h. The percentage (mean ± SEM) of G2/M cells is plotted versus cell type in the absence (solid bars) and presence of Cdt (cross-hatched bars); * indicates statistical significance (*p* < 0.05) when compared to untreated cells. Panel (**B**) shows the requirement for toxin containing active CdtB in order to induce cell cycle arrest (G2/M) in OKF6 (solid bars) cells and PGKs (cross-hatched bars). The percent (mean ± SEM) of G2/M cells is plotted against control cells containing no Cdt or cells receiving holotoxin containing PIP3 phosphatase active (CdtB^WT^ or the CdtB mutant subunit, CdtB^A163R^) or the PIP3 phosphatase inactive subunit mutants (CdtB^R117A^ or CdtB^R144A^). * indicates statistical significance (*p* < 0.05) when compared to untreated cells.

The ability of *Aa*Cdt to induce oral keratinocyte cell cycle arrest was first assessed on two immortalized cell lines, OKF6 and TIGK, which have been previously characterized [48,49], as well as PGKs [50]. Cell lines were exposed to 2 pg/mL (TIGK) or 10 pg/mL (OKF6) of Cdt, doses pre-determined to induce maximum accumulation of G2/M cells at 24 h (Figure 1A). TIGK cells exhibited an increase in the percentage of G2/M cells from 15.2 ± 0.14% (no Cdt) to 43.4 ± 4.20% in cells treated with Cdt; likewise, OKF6 cells treated with toxin exhibited an increase in the percentage of G2/M cells from 14.7 ± 0.5% (no Cdt) to 56.8 ± 2.3%. PGKs were exposed to 10 pg/mL Cdt for 72 h resulting in an increase in the percentage of G2/M cells from 18.0 ± 1.8% (no Cdt) to 34.2 ± 8.7%. As discussed earlier, we previously demonstrated that CdtB functions as a potent PIP3 phosphatase and further, the toxic effect on lymphocytes and other cell types was dependent upon this enzymatic activity [23,43,44,45]. To determine if Cdt-induced cell cycle arrest in oral keratinocytes was also dependent upon CdtB’s lipid phosphatase activity, we employed holotoxin containing one of the following CdtB subunits: fully active wildtype CdtB (CdtB^WT^), mutant CdtB^A163R^ which retains phosphatase activity, but is deficient in DNase activity or CdtB mutants (CdtB^R117A^ and CdtB^R144A^) that lack phosphatase activity but retain DNase activity [42]. As shown in Figure 1B, both OKF6 cells and PGKs exposed to 10 pg/mL Cdt containing enzymatically active CdtB^WT^ exhibited significant cell cycle arrest as determined by the accumulation of cells in the G2/M phase of the cell cycle. Similarly, cells treated with toxin containing the mutated subunit, CdtB^A163R^, retaining phosphatase activity also exhibited increases in G2/M cells: 46.1 ± 6.0% (OKF6) and 30.2 ± 8.0% (PGK). In contrast, OKF6 cells exposed to mutant CdtBs lacking phosphatase activity did not exhibit changes in the percentage of cells in the G2/M phase of the cell cycle: 13.4 ± 1.3% (CdtB^R117A^) and 14.0 ± 1.3% (CdtB^R144A^). PGK cells exposed to toxin containing these inactive CdtB mutant proteins also did not exhibit cell cycle arrest as the percentages of cells in the G2/M phase were 18.4 ± 3.2% (CdtB^R117A^) and 19.2 ± 3.0% (CdtB^R144A^). Similar results were observed with TIGK cells (data not shown).

**Figure 2:**
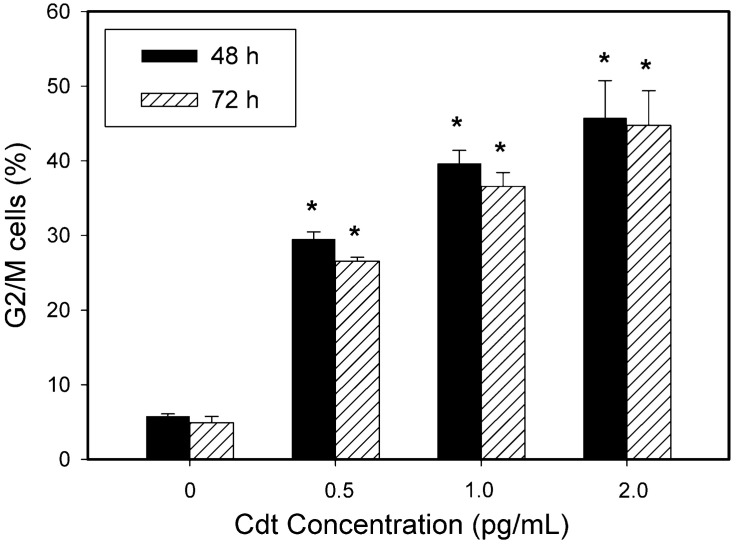
Cdt-induced accumulation of G2/M cells persists for 72 h. TIGK cells were treated with varying amounts of Cdt for 48 (solid bars) and 72 h (hatched bars). The percentage (mean ± SEM) of G2/M cells is plotted versus toxin concentration; * indicates statistical significance (*p* < 0.05) when compared to untreated cells.

As noted earlier, Cdt intoxication of proliferating cells typically leads to cell cycle arrest and eventually to cell death via apoptosis (reviewed in [3,22]). Our previous studies with lymphocytes demonstrated that Cdt-induced cell cycle arrest occurred within 16–24 h following exposure to toxin [51]. It is noteworthy that analysis of cell cycle arrest at later times (48 and 72 h) demonstrated a decrease in the accumulation of G2/M cells. This reduction was not the result of transient cell cycle arrest, but rather due to the onset of apoptosis which began after 24 h in the G2/M population and led to reduced levels of DNA and a concomitant reduction in propidium iodide fluorescence [51]. Thus, reduced propidium iodide fluorescence caused a shift of the arrested/apoptotic cells (G2/M) into what would normally be the S phase at 48 h and further decreases at 72 h shifted the cells into the G1/G0 population. To determine if oral keratinocytes responded to Cdt in a similar manner, we next performed cell cycle analysis of keratinocytes treated with Cdt for 48 and 72 h. As shown in Figure 2, TIGK cells treated with varying amounts of Cdt (0–2 pg/mL) exhibited a dose-dependent increase in the percentage of G2/M cells at 48 h: 29.5 ± 5.0% (0.5 pg/mL Cdt), 39.7 ± 4.4% (1.0 pg/mL Cdt) and 45.8 ± 4.3% (2.0 pg/mL Cdt). The percentages of cells in the G2/M phase did not significantly change at 72 h.

**Figure 3:**
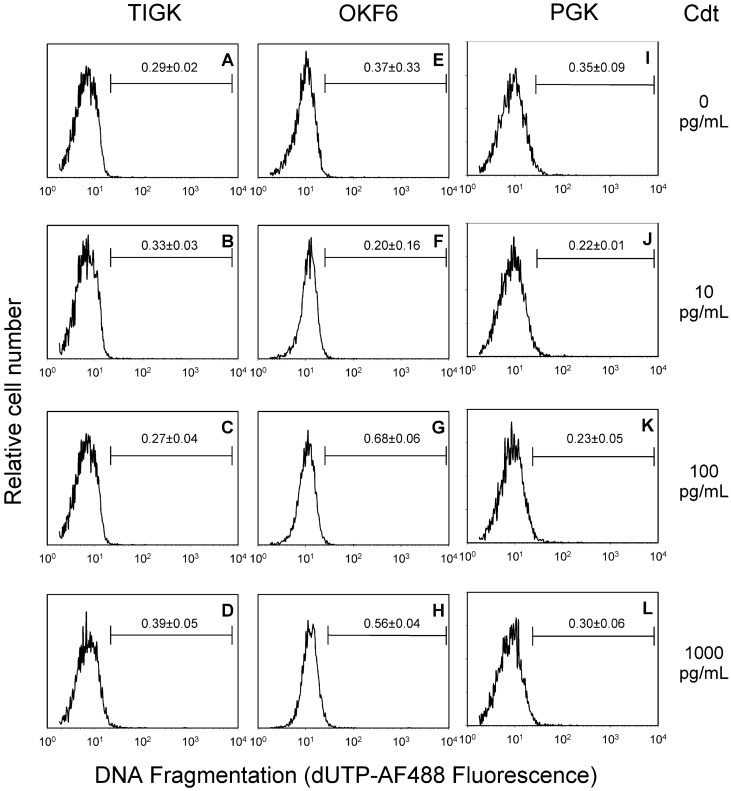
Cdt does not induce DNA fragmentation in oral keratinocytes treated with Cdt. The immortalized cell lines OKF6 and TIGK as well as PGKs were treated with 0–1000 pg/mL Cdt for 72 h and then analyzed for DNA fragmentation using the TUNEL assay. Individual panels show relative cell number plotted against DNA fragmentation (dUTP AFF488 fluorescence). Plots show representative results of three experiments; numbers represent the compiled results of all experiments: mean ± SEM of the percentage of cells containing dUTP fluorescence above that displayed in control cells. Bars represent gating area used to define positive fluorescence. Results from control cells are shown in panels (**A**) (TIGK), (**E**) (OKF6) and (**I**) (PGK); cells treated with 10 pg/mL Cdt are shown in panels (**B**) (TIGK), (**F**) (OKF6), (**J**) (PGK), cells treated with 100 pg/mL are shown in panels (**C**) (TIGK), (**G**) (OKF6), (**K**) (PGK) and cells treated with 1000 pg/mL are shown in panels (**D**) (TIGK), (**H**) (OKF6) and (**L**) (PGK).

**Figure 4:**
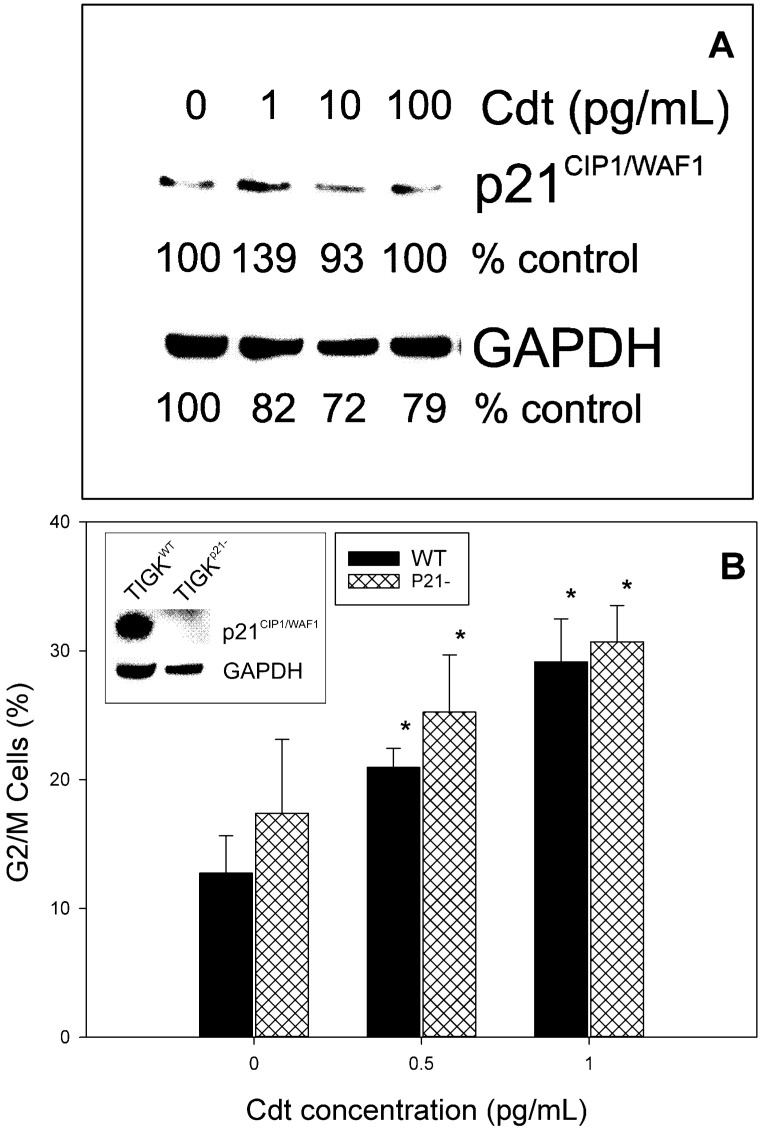
Cdt-induced cell cycle arrest does not involve the regulatory protein p21^CIP1/WAF1^. Panel (**A**) shows representative (3 experiments) results from TIGK cells treated with varying amounts of Cdt for 24 h. Cell lysates were monitored by Western blot for the levels of p21^CIP1/WAF1^; data are plotted as a percentage of p21^CIP1/WAF1^ levels observed in untreated cells versus Cdt concentration. GAPDH is shown as a gel loading control. Panel (**B**) shows results of cell susceptibility to Cdt-induced cell cycle arrest; CRISPR/cas9 gene editing was employed to produce a TIGK cell line deficient in p21^CIP1/WAF1^ expression (TIGK^p21-^). Inset shows the baseline levels of p21^CIP1/WAF1^ in both TIGK^WT^ and TIGK^p21-^ cells. TIGK^WT^ (solid bars) and TIGK^p21-^ (cross-hatched bars) cells were challenged with Cdt and monitored for the accumulation of cells in the G2/M phase of the cell cycle after 24 h. Results are plotted as the percentage (mean ± SEM) of G2/M cells versus Cdt concentration. * indicates statistical significance (*p* < 0.05) when compared to untreated cells. There was no statistical significance between TIGK^WT^ and TIGK^p21-^ cells at any concentration of toxin employed.

The results on cell cycle distribution in oral keratinocytes over time are clearly different from our previous observations with lymphocytes [51]. Moreover, these results suggest that Cdt exposure does not lead to loss of cellular DNA content after 24 h indicating that the toxin does not activate the apoptotic cascade in TIGK cells; similar results were observed for OKF6 (data not shown) and PGK cells which exhibited significant G2/M arrest at 72 h (Figure 1A). We next assessed oral keratinocytes, both cell lines and PGK, for their susceptibility to Cdt-induced apoptosis by analyzing DNA fragmentation (TUNEL assay). In these experiments toxin concentrations were employed that were equal to as well as several orders of magnitude greater than those doses required to induce cell cycle arrest. As shown in Figure 3, TIGK cells, OKF6 cells and PGKs exposed to 0–1000 pg/mL Cdt for 72 h failed to exhibit any evidence of DNA fragmentation. In previous studies, we also observed that lymphocytes exposed to Cdt exhibited a rise in p21^CIP1/WAF1^ levels and further, that this regulatory protein was required for apoptosis but not Cdt-induced cell cycle arrest [46]. Consistent with the inability of Cdt to induce apoptosis in oral keratinocytes was our finding that the toxin also did not induce an increase in p21^CIP1/WAF1^ (Figure 4A). Moreover, we employed CRISPR/cas9 gene editing to generate a TIGK cell line deficient in p21^CIP1/WAF1^ expression (TIGK^p21-^). As shown in Figure 4B, TIGK^p21-^ cells remained susceptible to Cdt-induced G2/M arrest as they exhibited dose dependent increases in the accumulation of cells in this phase of the cell cycle; the increases were similar and not statistically different from the increases observed with TIGK^WT^ cells.

**Figure 5:**
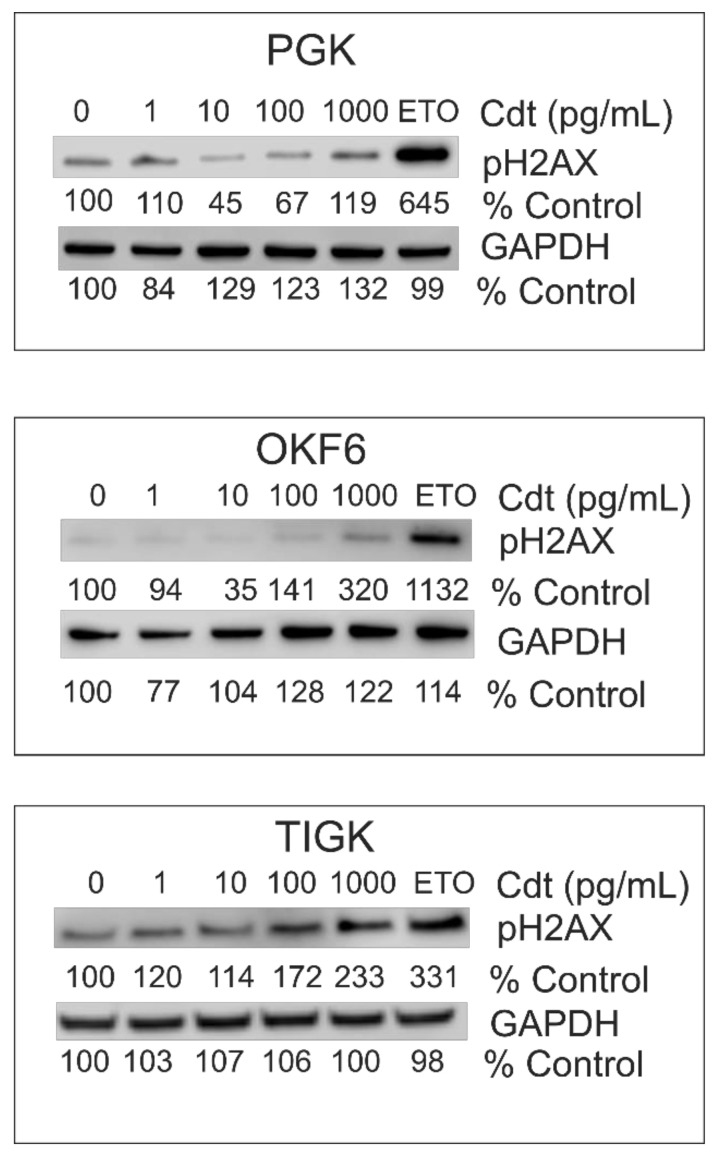
H2AX is not phosphorylated in oral keratinoyctes exposed to *Aa*Cdt. PGKs and immortalized cell lines OKF6 and TIGK, were challenged with varying amounts of Cdt (doses required to induce cell cycle arrest as well as higher doses) or 25 μM etoposide (ETO) for 4 h; cell lysates were assessed for pH2AX levels by Western blot. Results are representative of 3 experiments. Numbers indicate band intensity as a percentage of untreated cells. GAPDH is shown as a gel loading control.

Historically, the prevailing paradigm for Cdt’s mode of action involved DNA strand breaks due to CdtB-associated DNase activity (reviewed in [3,22]). Curiously, Cdt-induced strand breaks in mammalian cells have been difficult to demonstrate [3,22]. Instead, phosphorylation of the H2A histone family member X (H2AX) has been used as a surrogate for DNA damage and subsequent activation of the DNA damage response (DDR) [21,52,53,54,55,56]. Therefore, we next assessed Cdt for its ability to induce phosphorylation of H2AX (pH2AX) in oral keratinocytes. Results of these experiments are shown in Figure 5. PGKs did not exhibit increases in pH2AX at any Cdt concentration tested (0–1000 pg/mL) while the cells incubated with etoposide, a known inducer of DNA strand breaks and DDR activation [57] displayed >6-fold increase. OKF6 cells did not show increases in pH2AX at low concentrations of Cdt (1 to 100 pg/mL) sufficient to induce G2/M arrest, but did exhibit a 3-fold enhancement of the level of phosphorylated protein when exposed to 1000 pg/mL Cdt and >11-fold increase in the presence of etoposide. TIGK cells also failed to exhibit increases in pH2AX at the low levels of Cdt exposure (1 to 10 pg/mL) that lead to cell cycle arrest, whereas at higher concentrations, pH2AX levels increased 1.7-fold (100 pg/mL) and 2.3-fold (1000 pg/mL) as well as >3-fold in the presence of etoposide.

**Figure 6:**
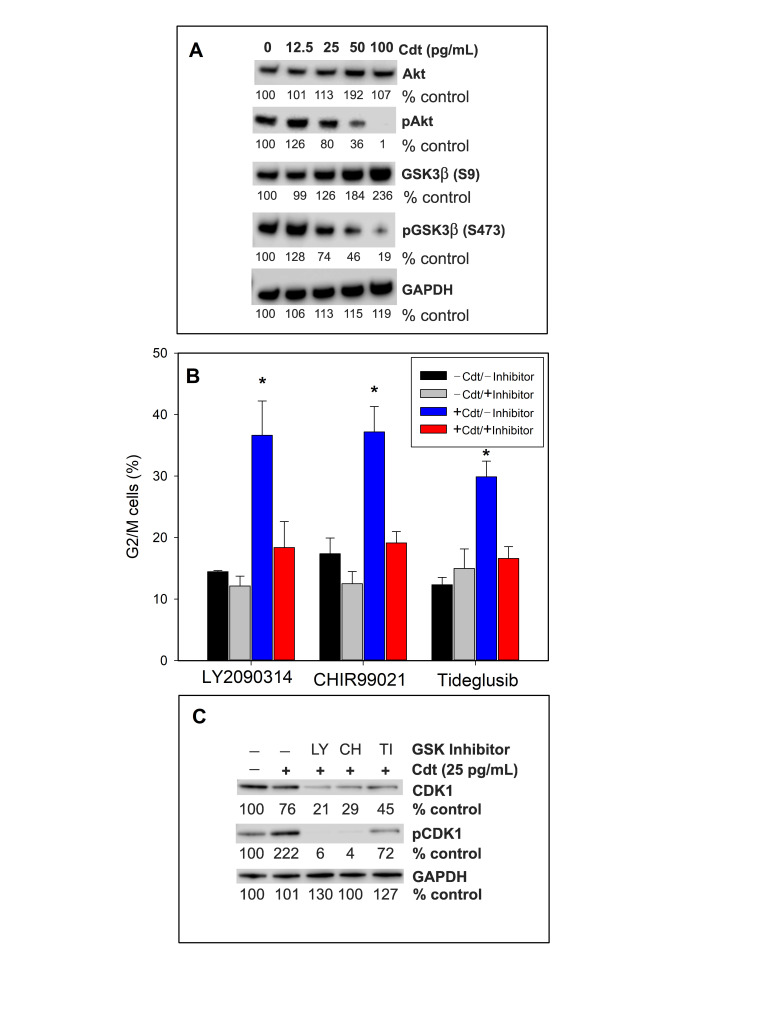
GSK3β activation is critical to Cdt-induced cell cycle arrest. Panel (**A**) shows representative results of PGKs treated with varying amounts of Cdt for 6 h. Cell lysates were monitored by Western blot for the levels of Akt, pAkt, GSK3β and pGSK3β. GAPDH is shown as a gel loading control. Results are representative of three experiments. Panel (**B**) shows the effects of TIGK pre-treatment with the GSK3β inhibitors, 10 μM LY2090314, 25 μM CHIR99021 or 25 μM Tideglusib for 60 min followed by the addition of Cdt (2 pg/mL). Cells were analyzed at 24 h for cell cycle arrest (G2/M accumulation). Data are plotted as the percentage (mean ± SEM) G2/M cells from three experiments; black bars represent untreated cells, grey bars cells exposed to inhibitor only, blue bars cells exposed to Cdt only and red bars cells pre-exposed to inhibitor followed by Cdt. * indicates statistical significance (*p* < 0.05) when compared to cells treated with Cdt only. Panel (**C**) shows the effect of three GSK inhibitors, LY2090314 (LY), CHIR99021 (CH) or Tideglusib (TI), on CDK1 and pCDK1 levels in TIGK cells. Cells were treated as described above and analyzed by SDS-PAGE and Western blot at 24 h. Representative (of four experiments) blots are shown; numbers represent the levels of protein expression as a percentage of control cell (untreated) content. GAPDH is shown as a gel loading control.

Activation of the G2/M checkpoint is often associated with DNA damage and activation of the DDR ultimately leading to inhibitory phosphorylation of cyclin-dependent kinase 1 (CDK1) thereby preventing cell progression into the M-phase of the cell cycle [58]. Failure to detect DDR activation in Cdt-treated oral keratinocytes led us to consider other possible mechanisms contributing to toxin-induced G2/M arrest. In this regard, our previous studies demonstrated that CdtB exhibits potent PIP3 phosphatase activity, similar to that of the tumor suppressors PTEN and SHIP [44]. Moreover, we have shown that human lymphocytes, mast cells and macrophages treated with Cdt exhibit PI-3K signaling blockade characterized by PIP3 depletion and reduced phosphorylation of Akt (inactivation) and GSK3β (activation) [23,42,43,45]. Likewise, PGK treated with increasing concentrations of Cdt (0–100 pg/mL) exhibited dose-dependent reductions in pAkt relative to control levels (Figure 6A): 80% (25 pg/mL), 36% (50 pg/mL) and 1% (100 pg/mL). Similarly, pGSK3β levels decreased to 74% (25 pg/mL), 46% (50 pg/mL) and 19% (100 pg/mL). Total Akt and GSK3β levels remained unchanged at lower doses of Cdt (12.5 and 25 pg/mL) while they increased at higher doses (50 and 100 pg/mL).

Exposure to Cdt not only leads to decreases in pGSK3β and, in turn, kinase activation, but we have also shown that Cdt’s toxicity on other cell types was dependent upon activation of this kinase [24,42,47]. As shown in Figure 6B, TIGK cells exposed to Cdt alone (blue bars), exhibited cell cycle arrest as the percentage of G2/M cells was significantly increased over control cell (no Cdt) values. In each instance, cells pre-treated with GSK3α/β inhibitors, LY2090314 and CHIR99021 or the GSK3β inhibitor, Tideglusib, followed by the addition of Cdt (red bars) displayed a small, yet statistically insignificant increase in the percentage of G2/M cells. Inhibitors alone (grey bars) did not alter the percentage of G2M cells. Thus, similar to lymphocytes, the toxic effect of Cdt on PGK is dependent upon GSK3β activation as inhibition of this kinase abrogates toxin-induced cell cycle arrest. 

As noted above, the critical event leading to G2/M cell cycle arrest and blockade of cell cycle progression into the M phase is the inactivation, i.e., phosphorylation, of CDK1 [59]. Therefore, we assessed the phosphorylation status of CDK1 in the presence of Cdt alone and in cells pre-treated with GSK3β inhibitors. As shown in Figure 6C, cells treated with Cdt alone exhibited >two-fold increase (222% of control values) in pCDK1 levels; total CDK1 levels were reduced by approximately 25%. In contrast, cells pre-treated with the three GSK3β inhibitors, LY2090314, CHIR99021 or Tideglusib, exhibited reduced phosphorylation to 6%, 4% and 72% of control values; interestingly, the inhibitors also reduced total CDK1 levels to 21%, 29% and 45%, respectively, of the levels observed in untreated cells.

## 3. Discussion

The diverse group of bacteria that are unique for their production of Cdts are also responsible for chronic infection and inflammatory disease affecting mucocutaneous tissue [3,22,60]. Thus, epithelial cells represent a potential host target of the toxin as they likely come in contact with Cdt and/or Cdt-producing bacteria. In addition to epithelial cells, other cells have been shown to be susceptible to Cdt; these include fibroblasts, macrophages and lymphocytes, among others [33,34,43,61,62,63,64]. While cells vary in their sensitivity to Cdt as well as toxic outcomes, the effect on most proliferating cells involves cell cycle arrest and eventually apoptosis. Historically, it has been suggested that this is the result of DNA damage and activation of the DDR, presumably due to CdtB-associated DNase activity [22,65,66]. Interestingly, we have identified an alternative mode of action for this toxin which is operative in human lymphocytes and other cells [23,43,44,67]. Specifically, we have demonstrated that *Aa*CdtB, *Hd*CdtB and *Cj*CdtB exhibit potent PIP3 phosphatase activity, thereby depleting cells of this crucial signaling lipid; this in turn leads to blockade of the PI-3K signaling pathway characterized by decreased phosphorylation of Akt (inactivation) and GSK3β (activation). Moreover, we have shown that these early events are linked to induction of G2/M arrest and apoptosis in lymphocytes that occur in response to very low doses of Cdt (0–50 pg/mL). 

The purpose of this study was to advance our understanding of the underlying mechanisms involved in *Aa*Cdt-mediated toxicity in oral epithelial cells and to determine whether these cells are intoxicated via a similar mode of action as that observed for lymphocytes. We employed two immortalized oral epithelial cell lines for this study and corroborated many of our findings with PGKs. All three cells were found to be sensitive to *Aa*Cdt-induced cell cycle arrest as toxin-treated cells not only accumulated in the G2/M phase within 24 h of exposure, but cell cycle arrest was induced at very low Cdt concentrations (≤10 pg/mL). Our results also demonstrated that the effects of Cdt on oral keratinocytes were dependent upon CdtB-associated PIP3 phosphatase activity as only toxin containing the active subunit, CdtB^WT^, as well as the CdtB^A163R^ mutant that retains PIP3 phosphatase activity, were capable of inducing cell cycle arrest. Toxin comprised of mutant CdtB subunits lacking PIP3 phosphatase activity while retaining DNase activity were not toxic. Furthermore, toxin-treated cells exhibited PI-3K blockade including GSK3β activation; the requirement for the active form of this kinase in mediating toxicity was demonstrated by the ability of GSK3α/β inhibitors, LY2090314 and CHIR99021 and the GSK3β inhibitor, Tideglusib, to block Cdt-induced cell cycle arrest.

While the underlying mechanism leading to Cdt-induced cell cycle arrest in oral keratinocytes involves CdtB-mediated PI-3K blockade as observed for other cell types, surprisingly, apoptosis was not a feature of Cdt-induced keratinocyte toxicity. Optimal doses of Cdt that induced cell cycle arrest in lymphocytes also result in apoptosis at 48 h [14]; exposure to higher doses of toxin accelerated the onset of apoptosis which could be detected as early as 24 h [51]. Using the TUNEL assay to evaluate DNA fragmentation, a late indicator of apoptotic death in association with caspase-3 activation revealed that none of the three oral keratinocytes tested exhibited evidence of Cdt-induced apoptosis at 24–72 h, regardless of the dose of toxin employed. Another observation indicating that oral keratinocytes are resistant to toxin-induced apoptosis is that cells remained suspended in the G2/M phase for at least 72 h, the longest time period measured. As noted earlier for lymphocytes, the onset of apoptosis led to DNA degradation, loss of DNA content and concomitant reduction in propidium iodide fluorescence. The net result of these events is that the apoptotic cells (initially the G2/M population) shifted to what appeared to be S phase cells at 48 h and with further DNA loss to G1/G0 cells at 72 h; this shift did not occur with toxin-treated oral keratinocytes. The transition of lymphocytes from cell cycle arrest to apoptosis was found to be dependent upon the cell cycle regulatory protein, p21^CIP1/WAF1^ [46]. We, and others, failed to detect an increase in p21^CIP1/WAF1^ following exposure of oral keratinocytes to Cdt [68]; this finding is consistent with the failure of Cdt to induce apoptosis in this type of cell. It is noteworthy that the TIGK^p21-^ cell line, deficient in p21^CIP1/WAF1^ expression, remained susceptible to Cdt-induced cell cycle arrest. This observation is similar to those reported in previous studies employing lymphocytes deficient in p21^CIP1/WAF1^ expression [46]. 

Our inability to detect Cdt-induced apoptosis in oral keratinocytes is different from the observations of Alaoui-El-Azher et al. [68]. It should be noted, however, that these authors exposed target cells to Cdt within the context of intact bacterial cells as opposed to the purified holotoxin used in the current study. Additionally, evidence for Cdt-dependent apoptosis was based upon use of an isogenic mutant lacking Cdt. Thus, it is entirely possible that in those studies, Cdt itself was not directly responsible for the induction of apoptosis. It is feasible that when Cdt is presented in the context of other bacterial cell-associated molecules it may serve as a co-factor for induction of apoptosis, yet by itself, is unable to activate the apoptotic pathway(s). Alternatively, Cdt may require additional factor(s) present in the bacterial cell in order to induce apoptosis in oral keratinocytes.

As noted earlier, several investigators have reported that Cdt-treated cells exhibit DDR activation exemplified by phosphorylation of H2AX; these findings have also been interpreted as indirect evidence of toxin-induced DNA damage due to CdtB-associated DNase activity [68,69,70]. However, we, and others, have demonstrated that the DDR is not activated (i.e., we do not observe increases in pH2AX) in lymphocytes under conditions of Cdt-treatment that involve exposure to optimal levels of toxin required to induce both cell cycle arrest and apoptosis [15,45,47,51]. Higher doses of toxin nominally increased pH2AX levels; however, these increases appeared to be the result of activation of the apoptotic cascade rather than a direct effect of Cdt on DNA damage [51]. We now demonstrate that under similar conditions, *Aa*Cdt does not induce increases in oral keratinocyte levels of pH2AX. These observations are consistent with *Aa*Cdt toxicity in lymphocytes; moreover, they are in line with our failure to detect apoptosis in oral keratinocytes. It should be noted that exposure to high levels of Cdt (>100 pg/mL) did result in small increases in pH2AX in the two immortalized cell lines, but not in PGK. Ohara et al. [71] also failed to detect pH2AX in periodontal tissue obtained from rats exposed to Cdt. In contrast, Alaoui-El-Azher and colleagues [68] reported on DDR activation in vitro using an oral keratinocyte cell line exposed to Cdt. As discussed above, those experiments employed exposure of an immortalized oral keratinocyte cell line to intact bacterial cells. Thus the differences with our observations may be related to the target cell employed and/or methodology as it relates to toxin exposure. Collectively, we draw the same conclusion from these results as we have from our studies with lymphocytes; activation of the DDR, and hence, DNA damage, are not a component of Cdt-induced cell cycle arrest in oral keratinocytes. In contrast, Cdts from other species have been shown to induce DDR activation in epithelial cells from non-oral tissues [38,72,73,74]. This raises the possibility that Cdts may utilize distinct mechanisms in various cell types; these differences could be related to requirements for higher doses of toxin to achieve toxicity [38,52,55,56]. Alternatively, the need for higher doses of toxin may result from the use of less pure Cdt preparations thereby introducing other entities of bacterial origin.

Our observations that Cdt-treated oral keratinocytes underwent cell cycle arrest in the absence of DDR activation is consistent with the observations of Sert et al. [15] who also demonstrated that HeLa cells exposed to Cdt exhibited cell cycle arrest (G2/M) under conditions that did not involve DNA damage. These observations go counter to the paradigm that activation of the G2/M checkpoint involves DNA damage and activation of the DDR ultimately leading to phosphorylation (inactivation) of CDK1 and failure of cells to progress into the M phase [58]. In this context, we did observe a significant increase in pCDK1 levels in cells treated with toxin alone; this led us to consider an alternative mechanism(s) contributing to phosphorylation of CDK1. Thus, we assessed the GSK3 inhibitors for their ability to impair CDK1 phosphorylation; indeed, cells treated with the inhibitors exhibited reduced pCDK1 levels that were below that observed in control cells. Moreover, the same cells also exhibited low levels of total CDK1 suggesting that GSK3β (and/or GSKα) contributes to both toxin-induced CDK1 phosphorylation as well as maintenance of CDK1 levels. In this context, it has been shown that GSK3 is capable of regulating protein stability by blocking or promoting proteasomal degradation [75,76]; protein levels shown to be regulated by GSK3 include those involved in cell proliferation, cell cycle progression, apoptosis and differentiation. Studies are in progress to further define this regulatory pathway as it relates to Cdt and possibly intoxication of other cell types.

The ability of Cdt to impair epithelial cell cycle progression, even in the absence of apoptosis, is significant as it relates to epithelium in general, and oral epithelium, in particular. Cell cycle arrest is likely to perturb epithelial turnover and eventually lead to altered barrier function. Indeed, there is growing evidence from studies employing human gingival explant models as well as in vivo animal models that demonstrate the ability of Cdt to disrupt the epithelial barrier and penetrate the epithelium causing cell cycle arrest [13,71,77]. Ohara et al. [71] observed that Cdt-mediated cell cycle arrest occurs in vivo within basal cells in the junctional and gingival epithelium; they further propose that cell cycle blockade contributes to subsequent desquamation and detachment of the junctional epithelium. It is now believed that initial colonization of supragingival biofilms by *A. actinomycetemcomitans* is not sufficient to cause periodontitis, but represents a risk factor for the onset of gingival inflammation. Now considered an accessory pathogen, the organism eventually translocates from the gingival margin through the gingival epithelium into the underlying connective tissue which is associated with conversion from health to disease [78,79,80]. In this regard, *A. actinomycetemcomitans* exhibits several virulence properties: tissue invasiveness, creation of a milieu that facilitates accumulation of other organisms, evasion of host defenses and the ability to promote inflammation; collectively, these contribute to the accumulation of inflammophilic organisms that mediate the downstream events in the pathogenesis of periodontitis (reviewed in [78,80,81]. We propose that Cdt is a contributing factor to several of these virulent properties of *A. actinomycetemcomitans* as it can function as a tri-perditious toxin and affect epithelial, lymphocyte and macrophage function thereby altering innate and acquired immunity as well as barrier integrity [3,82]. Cdt is able to exhibit these diverse effects and intoxicate multiple cell types by virtue of three unique properties that we have identified: (1) exploitation of a ubiquitous cell receptor, cholesterol [83,84,85]; (2) utilization of a novel host protein, cellugyrin, for cell entry and trafficking [86,87], and (3) its molecular mode of action is to disrupt PI-3K signaling, a pathway utilized by virtually all cells [23,43,44]. The results of these studies as well as those of the current report, advance our understanding of both the mechanisms that underlie Cdt toxicity of oral epithelial cells and the role of *Aa*Cdt in the pathogenesis of periodontitis. Furthermore, we propose that our observations may eventually lead to alternative approaches for treating this disease. Importantly, as the family of Cdt-producing organisms often target epithelial and mucosal surfaces, our current findings may be applicable to other Cdt-associated disorders involving nonoral tissue.

## 4. Materials and Methods

### 4.1. Oral Keratinocyte Isolation, Culture and Gene Editing

Primary gingival keratinocytes (PGK) were either obtained from ATCC (Manassas, VA, USA) or isolated from discarded healthy gingival tissue obtained from patients undergoing crown lengthening procedures as previously described [50]. Briefly, tissue was washed in HAMS F12 nutrient mixture [Gibco (Thermo Fisher Scientific, Waltham, MA, USA)] containing 1% penicillin/streptomycin and 1% ampthotericin and then cut into 0.3 cm^2^ fragments. The pieces of gingiva were incubated in HAMS F12 medium described above and containing 2% dispase (Sigma Aldrich Co.; Burlington, MA, USA) for 24 h at 37 °C. Tissue was then separated with vigorous pipetting in 0.05% trypsin/EDTA (Gibco) and the cell suspension was centrifuged, resuspended and incubated in Keratinocyte serum free medium (K-SFM; Gibco) containing bovine pituitary extract (BPE) (5 ng/mL), epidermal growth factor (10 μg/mL) and 2% penicillin/streptomycin.

The OKF6/TERT-2 cell line was established from oral mucosal epithelium (floor of the mouth) and immortalized by forced expression of telomerase using retroviral transduction (kindly provided by J. Rheinwald) [48]. OKF6 cells were incubated in K-SFM medium supplemented as above. The TIGK cell line was established from human gingival epithelial cells and immortalized with bmi1-transduction followed by human telomerase reverse transcriptase (hTERT) (kindly provided by RJ Lamont) [49]. TIGK cells were incubated in DermaLife K Basal Medium (Lifeline Cell Technology, Frederich, MD, USA) supplemented with glutamine, extract P, epinephrine, rh TGFα, hydrocortisone hemisuccinate, rh insulin, apo-transferrin and calcium chloride.

We employed pLentiCRISPR V2 to generate p21^CIP1/WAF1^-deficient TIGK cells (TIGK^p21-^) [88]. Briefly, three separate CRISPR-Cas9 guide sequences for p21^CIP1/WAF1^ were inserted into plentiCRISPR V2 plasmids with puromycin resistance replaced with a neomycin resistance cassette (CCATTAGCGCATCACAGTCG, ATTTCTACCACTCCAAACGC, CGACTGTGATGCGCTAATGG) (Genscript, Piscataway, NJ, USA). Plasmids were co-transfected into HEK-293T cells and viral supernatants collected for introduction into cells. Virus was added to cells at a MOI of 4 and incubated overnight; cells were washed, then incubated for an additional 24 h before selecting with 250 ng/mL Neomycin. Cells were plated for single cell cloning and p21^CIP1/WAF1^-negative lines generated from selected clones.

### 4.2. Preparation of Cdt

Construction and expression of the plasmid containing the *cdt* genes for the holotoxin (pUCAacdtABC^his^) as well as those constructs containing CdtB mutations have previously been reported [89]. The histidine-tagged holotoxin was isolated by nickel affinity chromatography as previously described [26].

### 4.3. Assessment of Cell Cycle Distribution and Apoptosis

To measure Cdt-induced cell cycle arrest, cells (1 × 10^6^) were incubated for 24 h, harvested following trypsin digestion, washed and fixed for 60 min with cold 80% ethanol [63]. Cells were stained with 10 µg/mL propidium iodide containing 1 mg/mL RNase (Sigma Aldrich Co.) for 30 min. Samples were analyzed on a Becton-Dickinson LSRII flow cytometer (BD Biosciences; San Jose, CA, USA); a minimum of 15,000 events were collected for each sample; cell cycle analysis was performed using Modfit version 5.0.9 (Verity Software House; Topsham, ME, USA). See Figure S1 for gating strategy and identification of cells in the G0//G1, S and G2/M phases of the cell cycle.

Cdt-induced apoptosis was assessed following 24, 48 or 72 h incubation in the presence of medium or Cdt. DNA fragmentation was measured using the TUNEL assay [In Situ Cell Death Detection Kit; (Sigma Aldrich Co.)] [14]. Cells were harvested as above and re-suspended in freshly prepared 4% formaldehyde and permeabilized with 0.1% Triton X-100 for 2 min at 4 °C. The cells were then washed with PBS and incubated in a solution containing fluorescein isothiocyanate (FITC)-labeled nucleotide and terminal deoxynucleotidyl transferase (TdT) according to the manufacturer’s specifications. FITC fluorescence was assessed by flow cytometry using a laser at 488 nm to excite the fluorochrome; emission was measured through a 530/30 nm bandpass filter.

### 4.4. Western Blot Analysis

Cells were treated as described and solubilized in 20 mM Tris-HCl buffer (pH7.5) containing 150 mM NaCl, 1 mM EDTA, 1% NP-40, 1% sodium deoxycholate and protease inhibitor cocktail (ThermoFisher Scientific). Samples (30 μg) were separated on 12% SDS-PAGE and then transferred to PVDF membranes. The membrane was blocked with BLOTTO and then incubated with one of the following primary antibodies for 18 h at 4 °C [7]: anti-Akt, anti-pAkt (S473), anti-GSK3β, anti-pGSK3β (S9), anti-GAPDH (Cell Signaling Technology; Danvers, MA, USA), anti-p21^CIP1/WAF1^ (Abcam; Cambridge, MA, USA), or anti-CDK1 and anti-pCDK1 (Thr14,Tyr15) (Invitrogen). Membranes were washed and incubated with goat anti-rabbit immunoglobulin conjugated to horseradish peroxidase (Southern Biotech Technology; Birmingham, AL, USA). The Western blots were developed using chemiluminescence and analyzed by digital densitometry (Li Cor Biosciences; Lincoln, NE, USA) as previously described [43].

### 4.5. Statistical Analysis

Mean ± standard error of the mean was calculated for replicate experiments. Significance was determined using a Student’s *t*-test with SigmaPlot Software version 14 (Systat; San Jose, CA, USA); a *p*-value of less than 0.05 was considered to be statistically significant.

## Data Availability

Data sharing not applicable as no datasets have been generated or analyzed in this study.

## Supplementary Materials

The following supporting information can be downloaded at: https://www.mdpi.com/article/10.3390/ijms231911831/s1. Figure S1 shows the gating strategy for defining cells in each of the phases of the cell cycle based upon propidium iodide (PI) fluorescence: G0/G1, S and G2/M. Figure S1A: shows the cell cycle profile for control (0 Cdt) TIGK cells. Figure S1B: shows the cell cycle profile for Cdt-treated (2 pg/mL) TIGK cells.
